# Many Neglected Tropical Diseases May Have Originated in the Paleolithic or Before: New Insights from Genetics

**DOI:** 10.1371/journal.pntd.0001393

**Published:** 2012-03-27

**Authors:** Gabriel Trueba, Micah Dunthorn

**Affiliations:** 1 Instituto de Microbiología Universidad, San Francisco de Quito, Quito, Ecuador; 2 Department of Ecology, Faculty of Biology, University of Kaiserslautern, Kaiserslautern, Germany; London School of Hygiene & Tropical Medicine, United Kingdom

## Abstract

The standard view of modern human infectious diseases is that many of them arose during the Neolithic when animals were first domesticated, or afterwards. Here we review recent genetic and molecular clock estimates that point to a much older Paleolithic origin (2.5 million years ago to 10,000 years ago) of some of these diseases. During part of this ancient period our early human ancestors were still isolated in Africa. We also discuss the need for investigations of the origin of these diseases in African primates and other animals that have been the original source of many neglected tropical diseases.

## Introduction

A prevailing view of the origins of modern human–specific infectious diseases is that many of them arose and spread during the advent of animal domestication and urbanization in the Neolithic or afterwards [1]–[3]. There is indeed evidence for Neolithic origins in such diseases as measles [4]. One consequence of this view is that the search for the origins of diseases, such as tuberculosis, malaria, pertussis, etc., has focused on domesticated animals and environments outside of Africa.

With new genetics and molecular clock data we are now beginning to understand that some neglected tropical diseases arose much earlier in the Paleolithic, such as tapeworm [5] or mycobacterial infections [6]. During this time, our hominid ancestors were still isolated in Africa [7]. Given these alternative, and much older, origin hypotheses, we propose that extensive research is needed in tropical environments in Africa. In this manuscript we will focus mainly on the origins of some neglected tropical diseases.

## Newer Molecular Methods and Older Potential African Origins

While quite uncommon now, leprosy was first recorded in humans around 600 BC in India [8]. Based on historic documents, it is has been thought that this disease was later brought to Europe during Greek military campaigns [8]. In support of this recent origin theory is an absence of leprosy in pre-Columbian Americans [8], and little genetic variation among isolates of *Mycobacteria leprae* [9], [10], the causative agent of this infectious disease. By contrast, phylogeographic and single nucleotide polymorphism (SNP) analyses point to *M. leprae* originating in Africa during the Paleolithic [10], [11]. This ancient date suggests that the current presence of little genomic variation may be due to a recent bottleneck [10], [11], possibly due to *M. leprae*'s low rate of infection [12]. This low infection rate could also explain the absence of leprosy in pre-Columbian Americans, even though their ancestors may have themselves been infected. In support of the SNP analyses, a molecular clock analysis suggests that the ancestor of *M. leprae* diverged from tubercle bacilli around 66 million years ago (MYA) [9], [11], prior to the origins of the genus *Homo* 2.5 MYA [7]. Analysis of non-synonymous nucleotide substitutions suggests that *M. leprae* underwent genomic decay between 10 and 20 MYA [9], [11].

Other diseases whose origins have been subjected to major debates are treponematoses, which include syphilis (*Treponema pallidum* subsp. *pallidum*), bejel (*T. pallidum* subsp. *endemicum*), yaws (*T. pallidum* subsp. *pertenue*), and pinta (*T. pallidum* subsp. *carateum*) [13]. While most of the debate focuses on the origins and spread of *Treponema pallidum* subsp. *pallidum*, recent phylogenetic and SNP analyses of treponemal genes suggest that the Old World *T. pallidum* subsp. *pertenue* is the oldest lineage [14]. Typical yaws-like lesions have been found in prehistoric human bones and hominids, indicating a Paelolithic origin of treponematosis [15]. The change from casual to venereal route of transmission in *Treponema pallidum* subsp. *pallidum* remains a puzzle. *Neisseria gonorroheae*, another venereal pathogen, may have evolved from a linage of *Neisseria meningitides* (upper respiratory tract inhabitant) during the Neolithic [16], and it may be related to the emergence of large villages.

*Bordetella pertussis*, the etiologic agent of whooping cough, was thought to have originated recently from *Bordetella bronchiseptica* that was infecting domestic animals such as pigs and dogs [1]–[3]. Although analysis of DNA sequences of multiple loci (MLST) indicated that *B. pertussis* evolved from *B. bronchiseptica* [17], recent molecular clock estimations suggest that the divergence time between *B. pertussis* and *B. bronchiseptica* associated with domestic animals is 1.1 and 5.6 MYA [17] before the origin of the *Homo sapiens* 0.2 MYA [7]. Genomic decay in *B. pertussis* may have been the result of evolution among ancestral hominids and adaptation to these hosts [17], [18]. Additionally, human strains of *B. parapertussis* (a bacteria causing less severe whooping cough in humans) diverged from animal *B. bronchiseptica* 0.7 to 3.5 MYA and have evolved from a different clade than *B. parapertussis* isolated from domestic animals [17]. Therefore, molecular data suggest that *B. pertussis* and *B. parapertussis* originated far earlier than the Neolithic period and did not originate in domestic animals [17].

Abundant problems exist in inferring phylogenetic relationships [19]; these problems are further exacerbated in molecular clock analyses that attempt to date these relationships [20]–[22]. While keeping these potential methodological problems in mind, recent molecular clock analyses, as well as other genetic investigations using single nucleotide polymorphisms and phylogeography, do tentatively suggest that some of our modern human infectious diseases did not arise with the advent of animal domestication in the Neolithic as previously thought. Rather, these diseases—such as tuberculosis, leprosy, and treponematosis—have a much older origin in the Paleolithic. During this ancient time our hominid ancestors still may have been living in Africa. A deeper understanding of the origins of these diseases and possibly others, then, will require us to investigate them in African primates and other animals.

## A Need for Research in African Primates and Other Animals

Most research on the African origins of human infectious diseases focuses on HIV and malaria. Nevertheless, there are some initial studies in other diseases, such as *M. leprae* being found in primates showing signs of leprosy [23], [24]. The significance of non-human primate leprosy is unknown because of a lack of genetic information on the etiologic agents. At this point it is not possible to decipher if these primates, like armadillos [24], contracted their infections from humans, or if they were the original source of this modern human infectious disease. While it is unknown whether this leprosy from non-human primates could be passed to humans, there is some evidence that leprosy from armadillos is zoonotic [25].

Similarly, *T. pallidum* subsp. *pertenue's* infection rates are high in both humans and primates in yaws-endemic areas of West Africa [14]. A simian yaws-like skin disease caused by a variant closely related to the human *T. pallidum* subsp. *pertenue* [14], [26] that does not appear to be the result of recent cross infection from humans has been described [14]. It has been shown that inoculation with the simian strain can cause a yaws-like infection in humans, suggesting that cross species transference is also possible [14]. Additionally, *T. pallidum* subsp.*pertenue* is reported to cause genital ulcerations in African primates [27]. These data suggest that skin treponematosis may have evolved within African primates and our own ancestral human species.

**Figure 1:**
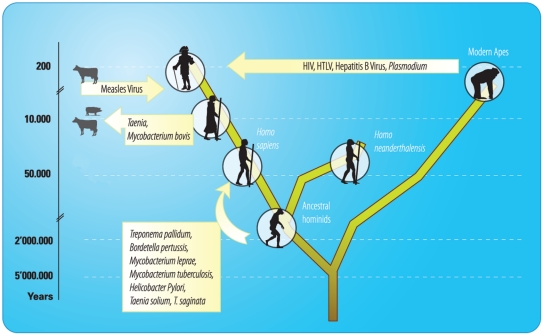
Origins of human-specific infectious diseases. Arrows indicate suggested direction of the transmission.

Pathogen crossing of host species barriers is a common occurrence in natural environments (zoonosis and anthroponosis). However, acquiring traits that enable efficient transmission within a given host species is a more unusual event. Adaptations to new hosts seem to occur more frequently in pathogens infecting phylogenetically related hosts [28]. The recent evolution of human-specific pathogens such as hepatitis B virus [29]–[31], HIV [32], human T cell lymphotropic virus (HTLV) [33], and malaria [34] from African primates follows this pattern. Other pathogens, such as *M. leprae*, *M. tuberculosis* [6], *B. pertussis*, *B. parapertussis*, *Treponema pallidum*, herpesviruses [35], papillomaviruses [36], *Helicobacter pylori* [37], *Taenia solium*, *T. saginata* [5], and even human intestinal microbiota [38], may have coevolved in ancestral hominids in Africa. Close contact with *Homo neantherthalensis* [39], or other archaic humans, may have also played a role in the introduction of some of these infectious diseases to modern humans (Figure 1). This evolutionary adaptation to a specific host transmission may be accompanied by a trade-off that reduces the competence to cross host species barriers and may involve genome decay [18].

Despite the recent evidence that many human infectious diseases have originated in primates, the study of infectious diseases of primates (especially apes) and other African animals is still a neglected field of research. African primates, including human's closest relatives the chimpanzee and the bonobo, are not only genetically similar to humans, but they also share the same habitats and food as humans in many regions of central Africa. Additionally, many people in this region consume ape meat and are exposed to blood and fluids from these animals [33]. Therefore, African primates remain an untapped source of information required to complete the puzzle of the mechanisms of origin and evolution of many human pathogens. As the genomic data from a wider population of pathogens and microbiota of humans and other animals become available, we will have a better understanding of the distribution of microbial pathogens and commensals and the mechanisms that govern transmission among different animal species. The discovery of the factors involved in crossing the host species barrier and the evolution of human-specific pathogens may help the identification of human activities that can potentially promote the emergence of new infectious diseases. Finally, many of these tropical ancient infections may have caused high mortality and contributed to human evolution, especially the shaping of the human immune system for longer periods of time than previously thought.

**Key Learning Points**

- Unlike the prevailing view, many human-specific infectious diseases may have originated in the Paleolithic period.
- During the Paleolithic period, many human-specific infectious diseases may have originated in primates, not in domestic animals.
- Crossing the animal species barrier, and evolving to be a host-specific pathogen, is facilitated by phylogenetic relatedness between the host species acting as a pathogen's source and the host species acquiring the new pathogen.
